# Response of Qualitative Traits and Antioxidant Systems to Chitosan Postharvest Treatment in ‘Black Golden’ Japanese Plum

**DOI:** 10.3390/foods11060853

**Published:** 2022-03-17

**Authors:** Giuseppina Adiletta, Giovanna Gliottone, Marisa Di Matteo, Milena Petriccione

**Affiliations:** 1 Department of Industrial Engineering, University of Salerno, Via Giovanni Paolo II, 84084 Fisciano, Italy; gadiletta@unisa.it; 2 Council for Agricultural Research and Analysis of Agricultural Economics (CREA), Research Center for Olive, Fruit and Citrus Growing, Via Torrino 3, 81100 Caserta, Italy; giovannag88@hotmail.it (G.G.); milena.petriccione@crea.gov.it (M.P.)

**Keywords:** Japanese plum, antioxidant systems, cold storage, edible coating

## Abstract

In this study, the influence of a chitosan-based coating was assessed on qualitative traits and non-enzymatic and enzymatic antioxidant systems in *Prunus salicina*, ‘Black Golden’ Japanese Plum, during 35 days of cold storage followed by 3 days at 24 °C to evaluate its shelf life. Chitosan coating delayed the physico-chemical traits such as the total soluble solids, titratable acidity, firmness and skin color associated with the plum ripening process during cold storage and shelf life. Furthermore, the highest bioactive compounds’ content and antioxidant activity in the coated plum were registered during the stored period. Chitosan-based coating enhanced the activities of superoxide dismutase and ascorbate peroxidase during cold storage and shelf-life. Moreover, this edible coating contributes to reducing membrane damages with lower lipoxygenase activity and, consequently, malondialdehyde accumulation. A multivariate statistical analysis approach identified the two key components, correlated to all analyzed traits, that influenced the changes within chitosan coated and uncoated Japanese plums during storage. Chitosan coating is a cheap and eco-friendly tool to delay ripening process and to improve the antioxidant systems and storability of the ‘Black Golden’ Japanese plum.

## 1. Introduction

Japanese plum (*Prunus salicina* Lindl.) is an important stone fruit species cultivated worldwide, with a global production that reaches over 12 million tons [1]. Fresh plums are appreciated by consumers for their good taste related to a balance between soluble solids’ content and acidity as well as for their health promoting potential due to high nutraceutical compounds’ content’ mainly phenolics, carotenoids, vitamin C and fiber [2,3,4]. The main challenge to the plum fruit industry is to maintain the nutritional value along the supply chain, avoiding huge economic losses due to poor postharvest management practices [5]. The quantity of postharvest losses should be halving, as well as food losses along the production and supply chains by 2030, as established by the European Commission that has recently launched a plan that supports the UN [6]. Several studies have demonstrated that the Japanese plum shows climacteric as well as non-climacteric ripening patterns, depending on the cultivar or genotype [7,8]. Fresh plum fruit have a relatively limited shelf life, consequently the acquisition of knowledge about the postharvest physiology of Japanese plum and the application of new postharvest technologies have been the key to maintain and extend the shelf life of this perishable fruit.

Edible coatings represent green, alternative and sustainable strategies that improve the postharvest life of whole and minimally processed fruit. Several compounds and antioxidant substances as well as alginate, chitosan, pectin, carbohydrates, sorbitol, carboxymethylcellulose, beeswax, grease, shellac resin and starch were used to realize edible coatings in plum fruit [9,10,11,12,13].

Chitosan is widely used to make edible coatings due to its biocompatibility, biodegradability, low toxicity, easy availability, low cost, film-forming properties and antimicrobial action [14,15]. Whilst chitosan-based coating is widely applied in several fruit crops as a postharvest preservative technique, in literature only a few studies were found on chitosan coated plum fruit. Kumar et al. [16] tested a chitosan-based coating (2%) on the storage life and quality of ‘Santa Rosa’ plums, demonstrating a slowdown in qualitative decay and an extension of the storage life of the plums up to 35 days under cold storage. Furthermore, the combined effects of chitosan (1%) and ascorbic acid (40 mM) improved the plums’ (cv. ‘Sanhuali’) fruit quality at 5 °C for 20 days [17].

In this study, we evaluated the influence of chitosan-based coating on the physico-chemical traits in *P. salicina*, ‘Black Golden’ Japanese plum during 35 days of cold storage followed by 3 days at 24 °C, to evaluate the shelf life. Furthermore, the changes in non-enzymatic and enzymatic antioxidant systems combined with oxidative damages during cold storage and shelf life were investigated.

## 2. Materials and Methods

### 2.1. Fruit Samples and Experimental Design

‘Black Golden’ Japanese plums were randomly collected at the commercial ripening stage from an experimental orchard grown under standard commercial practices in Pignataro Maggiore (Caserta, Southern Italy; 41°09′ N, 14°08′ E), owned by the CREA Center of Research For Olive, Fruit and Citrus Crops (Caserta, Southern Italy). Fruit were transported to the laboratory and screened for uniform size and the absence of defects or decay.

Fruit were randomly distributed into two lots (approximately 250 fruit in each lot). Chitosan solution with 90% deacetylation and a molecular weight of 360 kDa at 1% (*w/v*) was realized as described by Adiletta et al. [18]. Chitosan concentration (1%) of coating has been chosen after preliminary test using 0.5, 1 and 2% (*w/v*) chitosan concentrations (data not shown). Fruit were stored at 2 °C and 95% relative humidity and were analyzed every week until 35 days of cold storage (CS) and subsequently they were evaluated after 3 days of shelf life (SL) at 24 °C Uncoated fruit were dipped in distillate water. Five biological replicates per treatment (eight fruit per replicate) for each sampling date were prepared. Three replicates were used to assess the fruit properties during cold storage, while the other two were used for testing the SL. All analyses were assessed at harvest, at each sampling date and after SL.

### 2.2. Physico-Chemical Traits

Fruit weight loss was determined at each sampling date and expressed as the percentage loss of initial weight [19]. Total soluble solid content (TSS, °Brix) was measured in the flesh juice by digital refractometer (Sinergica Soluzioni, DBR35, Pescara, Italy), while titratable acidity (TA) was determined by titrating 10 mL of flesh juice with 0.1 N NaOH and expressed as g malic acid per L juice [20]. Fruit color parameters were measured using Minolta colorimeter (CR5, Minolta Camera Co., Osaka, Japan) and color changes were reported as lightness (L*); chroma (C*); and hue angle (H°) [21]. A digital penetrometer (TR Snc., Forlì, Italy) equipped with an 8-mm probe, was used to test for fruit firmness, expressed in Newton (N).

### 2.3. Bioactive Compounds and Antioxidant Activity

Total phenolics, anthocyanins, flavonoids and antioxidant activity were determined using methanolic extract completed in accordance with Martínez-Esplá et al. [7]. Fruit flesh tissues (5:10 *w/v*) were homogenized with methanol (80% *v*/*v*) containing 2 mM NaF; the extract was centrifuged at 15,000× *g* for 15 min at 4 °C. The Folin–Ciocalteu method was used to quantify the total phenolics (TP) using gallic acid as standard and results were expressed as mg gallic acid equivalent (GAE) per 100 g fresh weight (FW) [22]. The total anthocyanins’ content (TAC) was estimated by the pH differential method in agreement with Adiletta et al. [23] and the results were expressed as cyanidin-3-glucoside equivalents (C3G) per 100 g FW. The aluminum chloride colorimetric method was used to determine the total flavonoid content (TF), using catechin as standard [24]. The results were expressed as milligrams of catechin equivalent (CE) per 100 g fresh weight (FW). Total antioxidant activity (DPPH) was quantified by using DPPH assay and the results were expressed as µmol of Trolox equivalent (TE) per g fresh weight [23].

The ascorbic acid content (AA) of the plum fruit was determined following the method described by Petriccione et al. [25] with a mixture assay contained 400 µL extract, 0.3% (*v*/*v*) metaphosphoric acid and (5:1, *v*/*v*) diluted Folin’s reagent, in a final volume of 2 mL. The results were expressed as mg ascorbic acid (AA) per 100 g FW.

### 2.4. Enzyme Extraction and Enzymatic Activity Assays

Crude enzyme extract was prepared by homogenizing frozen fruit powder (1:3 *w/v*) in 100 mM potassium phosphate buffer (pH 7.8) containing 1 mM sodium EDTA (pH 7), 2 mM DTT, 1 mM PMSF, 0.2% Triton X-100, 5% (*w*/*v*) PVPP, and 5 mM ascorbic acid (the ascorbic acid must be used only for APX enzyme extraction), as reported by Adiletta et al. [26]. Total soluble proteins’ content was determined by the Bradford assay [27].

#### 2.4.1. Ascorbate Peroxidase and Superoxide Dismutase Activity

Ascorbate peroxidase (EC 1.11.1.11; APX) activity was evaluated according to Modesti et al. [28] monitoring the oxidation of ascorbic acid at 290 nm. The mixture assay consisted of 100 mM potassium phosphate buffer (pH 7), 0.33 mM ascorbic acid, 0.35 mM H_2_O_2_, 0.66 mM sodium EDTA (pH 7) and 150 µL of crude enzyme extract in a final volume of 1.5 mL. APX activity was expressed in µmol of ascorbate per mg protein. Superoxide dismutase (EC 1.15.1.1; SOD) activity was assessed as reported by Adiletta et al. [18] based on the photoinhibition on nitro blue tetrazolium (NBT) reduction. The reaction mixture consisted of 50 mM potassium phosphate buffer pH 7.8, 0.1 mM sodium EDTA (pH 7.0), 13 mM methionine, 75 µM NBT, 2 µM riboflavin and 600 µL of crude enzyme extract in a total volume of 1.5 mL. The reaction was started by adding riboflavin, and after 15 min of incubation at room temperature under continuous light, the absorbance at 560 nm was measured. SOD activity was expressed as Unit per mg protein, where one unit is the amount of enzyme that leads to 50% inhibition of NBT reduction.

#### 2.4.2. Guaiacol Peroxidase, Polyphenol Oxidase and Lipoxygenase Activity

Guaiacol peroxidase (EC 1.11.1.7; GPX) activity was assayed according to Modesti et al. [28]. The reaction mixture contained 100 mM potassium phosphate buffer pH 7, 0.20 mM sodium-EDTA pH 7.0, 4.0 mM H_2_O_2_, 6.4 mM guaiacol and 250 µL of crude enzyme extract in a final volume of 1 mL. Polyphenol oxidase (PPO) and lipoxygenase (LOX) activity were determined followed the method and the assay buffers described by Pasquariello et al. [29]. PPO activity was monitored at 398 nm using enzyme extract (100 µL) incubated with a buffered substrate (500 mM catechol in 100 mM sodium phosphate buffer pH 6.4) in a final volume of 1.5 mL. LOX activity was assayed in a reaction mixture contained 100 mM sodium phosphate buffer pH 6, 0.17 mM linoleic acid sodium salt, and 50 µL of crude enzyme extract in a final volume of 1.5 mL.

GPX, PPO and LOX activity were expressed as mmol per mg protein and µmol per mg protein, respectively.

#### 2.4.3. Malondialdehyde Content Determination

Malondialdehyde (MDA) content was measured by the method of Adiletta et al. [22]. Mixture assay contained 500 µL of the supernatant were mixed with 1.5 mL of 15% trichloroacetic acid containing 0.5% thiobarbituric acid. MDA was determined by measuring the adduct MDA-thiobarbituric acid. The MDA content was expressed as nmol per g FW.

### 2.5. Statistical Analysis

All results were expressed as the mean ± standard deviation. Statistical significance among chitosan-coated and uncoated plums was analyzed by one-way analysis of variance (ANOVA), and Duncan’s test at 5% level was calculated to compare the differences between means. Furthermore, statistical significance between storage times was evaluated. Differences at *p* < 0.05 were considered significant and were indicated with different letters. A principal component analysis (PCA) was applied to describe the influences of chitosan treatment on physico-chemical and antioxidant systems and to identify the components responsible for the main variations in the dataset. All analyses were performed using the SPSS^®^ software package, version 20.0 (SPSS Inc., Chicago, IL, USA).

## 3. Results

### 3.1. Effect of Chitosan Postharvest Treatment on Physico-Chemical Features

**Figure 1:**
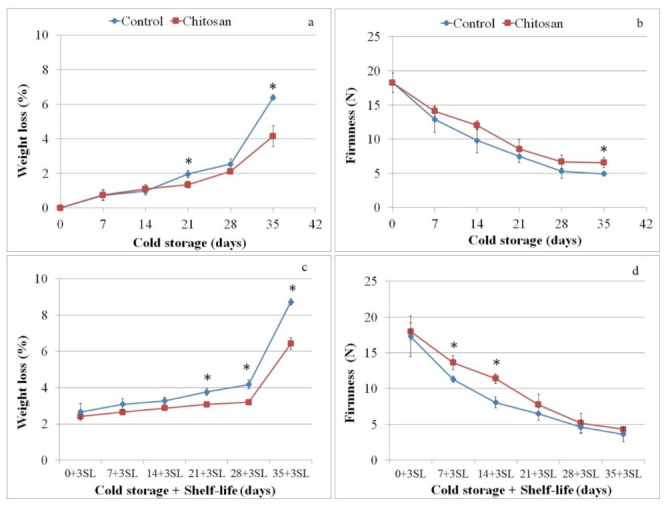
Weight loss (%) and firmness (N) at harvest (0) and after 7, 14, 21, 28 and 35 days of cold storage (**a**,**b**) followed by 3 days of shelf life (**c**,**d**) for chitosan-coated (Chitosan) and uncoated fruit (Control). One asterisk (*) indicates *p* value smaller than 0.05 (*p* < 0.05).

Cumulative weight loss showed higher values in the uncoated fruit compared to chitosan-coated fruit with progression of the storage period. After 35 days of cold storage uncoated and chitosan-coated fruit reached a maximum weight loss of 6% and 4%, respectively (Figure 1a). Chitosan coating limited the fruit weight loss compared with uncoated fruit during the shelf life with a significant increase in samples stored for 35 days at 2 °C and keeping the fruit at 24 °C for three days (Figure 1c).

Firmness values decreased under the cold storage conditions (Figure 1b) and after the shelf-life period (Figure 1d). At the end of cold storage conditions, coated fruit retained significant higher firmness compared to untreated ones, while no statistical difference in shelf life was registered.

Chitosan coating delayed the increase in TSS in coated plums compared to uncoated ones throughout the cold storage and shelf life (Table 1). Chitosan-coated fruit showed an increase of 15.04% and 19.00% during cold storage and subsequent shelf life, respectively, compared to uncoated fruit that showed an increment of 22.46% and 23.63% in the same period.

TA content displayed the same trends with a constant decrease in every sampling date (coated and uncoated fruit) during the storage and shelf-life period. TA loss was 44% and 57.08% in coated and uncoated fruit from harvest to the end of cold storage while after shelf life a further loss of 24.69% and 36.4% in the same samples was registered.

Results also showed significant effects of storage conditions and chitosan coating on color parameters such as L*, Chroma (C) and Hue angle (H). These parameters displayed a decrease at each sampling date during cold storage with significant differences among chitosan coated and uncoated samples.

### 3.2. Evaluation of Chitosan Coating on Bioactive Compounds and Antioxidant Activity

The content of polyphenols (TP) and flavonoids (TF) increased throughout cold storage and shelf life in all analyzed samples (Table 2). Chitosan-coated fruit exhibited an increase of TP and TF content at each sampling date during cold storage (Table 2). At the end of cold storage, chitosan coated fruit displayed the highest values in TP and TF content reaching values of 269.7 ± 14.0 mg GAE per 100 g FW and 115.7 ± 7.5 mg CE per 100 g FW, respectively. In particular, the same trend was registered during the shelf-life period with higher total phenolic and flavonoid content in coated fruit, but they showed lower values compared to cold stored samples at the same sampling date (Table 2). Coated samples after 35 days of cold storage combined with shelf-life conditions, detected a decrease of 13% and 15.4% in TP and TF content, respectively.

The content of anthocyanins and carotenoids increased in plum fruit during cold storage and shelf life but untreated fruit resulted in significantly higher values than the coated ones. Maintenance at room temperature led to a reduction of carotenoids’ content compared with the cold-stored sample with a higher percentage reduction after 35 days of cold storage plus 3 days at 24 °C.

In plums a decrease in ascorbic acid content has been observed but chitosan-treated fruit were generally characterized by higher values during cold storage and shelf life at room temperature.

Antioxidant activity increased until day 35 in both samples, with higher significantly differences at day 7 and day 14 plus 3 days of self-life in coated fruit compared with the uncoated fruit, respectively.

### 3.3. Effectiveness of Chitosan-Based Coating on Antioxidant Enzymes Activity

**Figure 2:**
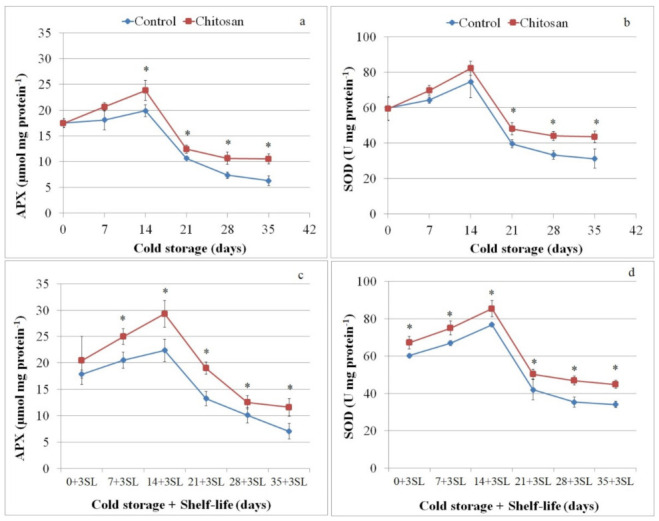
Ascorbate peroxidase (APX; µmol ascorbate per mg protein activity) and superoxide dismutase (SOD, U per mg protein) at harvest (0) and after 7, 14, 21, 28 and 35 days of cold storage (**a**,**b**) followed by 3 days of shelf life (**c**,**d**) for chitosan-coated (Chitosan) and uncoated fruit (Control). One asterisk (*) indicates *p* value smaller than 0.05 (*p* < 0.05).

Antioxidant enzymes involved in ROS detoxification in plum fruit throughout cold storage and shelf life are shown in Figure 2. The variation of SOD and APX activity was similar in all tested samples, but the enzymes’ activity was significantly higher in chitosan-coated fruit compared to uncoated ones, showing the highest values after 14 days of cold storage (Figure 2a,b). During shelf life, the variation of SOD and APX activities in all samples showed a similar pattern with higher values in the coated fruit (Figure 2c,d).

### 3.4. Effect of Chitosan Coating on Enzymatic Browning and Membrane Damage

Treatment with chitosan reduced the GPX activity significantly, showing an increase during first 14 days of cold storage followed by a decrease until the end of the experiment. The same trend of GPX activity has been observed during shelf-life period with higher values in uncoated fruit. PPO activity increased rapidly as storage time progressed mostly in untreated samples. Chitosan coating inhibited PPO activity by about 25% more than control after 35 days of cold storage. A slight increase in PPO activity was observed, maintaining the same gap between the coated and uncoated fruit at the end of shelf life.

During cold storage and shelf life the membrane damages in treated and untreated fruit were monitored by measuring LOX activity and MDA content (Table 3). LOX activity in plum fruit stored at 4 °C displayed a slight increase until 28 days and a rapid raise only appeared after 35 days. MDA content continuously increased during storage and content in uncoated samples remained significantly greater than coated fruit. During the shelf-life period, LOX activity and MDA content highlighted the same trend observed in cold-stored fruit with significantly higher values in uncoated fruit. However, the chitosan-based coating inhibited LOX activity and MDA content in plum fruit during cold storage and the shelf-life period.

Cell membrane permeability was evaluated by electrolyte leakage to monitor the physical injury to the cell membrane resulting from oxidative stress in chitosan-coated fruit during cold storage and shelf life (Table 3). Electrolyte leakage exhibited an uptrend in all tested samples with an increase in storage days, with a lower valuer in coated fruit. Electrolyte leakage of the uncoated fruit increased up to the highest levels (82 and 88%), which were 15% and 18% higher than the coated ones at the end of cold storage and shelf life, respectively.

### 3.5. PCA Analysis

**Figure 3:**
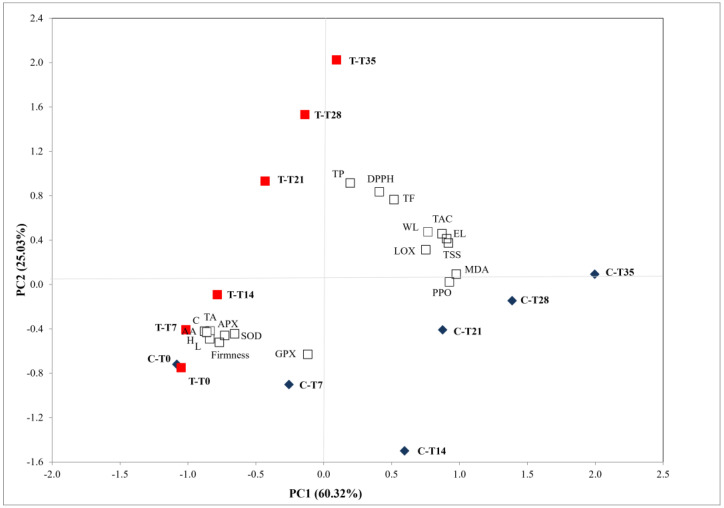
2D-principal component analysis plot of analyzed attributes in ‘Black Golden’ Japanese plum at harvest (C-T0: uncoated fruit; T-T0 chitosan-coated fruit 0), 7 (C-T7: uncoated fruit; T-T7 chitosan-coated); 14 (C-T14: uncoated fruit; T-T14 chitosan-coated); 21 (C-T21: uncoated fruit; T-T21 chitosan-coated); 28 (C-T28: uncoated fruit; T-T28 chitosan-coated); and 35 (C-T35: uncoated fruit; T-T35 chitosan-coated) days of cold storage (WL: weight loss; TSS: total soluble solid content; TA: total titratable acidity; Firmness; L: L* value; H: hue angle; C: Chroma; TP: polyphenol content; TF: flavonoid content; TAC: total anthocyanin content; AA: acid ascorbic content; DPPH: radical scavenging activity; EL: electrolyte linkage; APX: ascorbate peroxidase; SOD: superoxide dismutase; GPX: guaiacol peroxidase; LOX: lipoxygenase; PPO: polyphenol oxidase; MDA: malondialdehyde content).

PCA analysis was performed to pinpoint the influence of the chitosan treatment on the variations of physico-chemical and nutraceutical traits, antioxidant and browning enzymes in plum fruit during cold storage (Figure 3) and shelf life (data not shown). The total variability, analyzed by eigenvalues of the covariance matrix, was explained by two principal components that accounted for 85.35% (PC1 60.32% and PC2 25.03%) and 84.65% (PC1 61.18% and PC2 23.47%) of the total variance in the dataset during cold storage and the subsequent shelf life period, respectively.

WL, TSS, EL, TAC, MDA content, PPO and LOX were highly positively correlated with PC1 whereas firmness, TA, AA, APX, SOD and L*, C and H were negatively correlated. The variables positively correlated with PC2 were TP, TF, DPPH while GPX was negatively correlated (Figure 3). Chitosan-coated samples were noticeably separated along PC2 and were shifted from bottom-left to the left-top quadrant while uncoated ones separated along PC1 and were shifted from the bottom-left to bottom-right quadrant suggesting considerably changes in analyzed traits in coated and uncoated fruit. During the shelf-life period an additional shift along the positive PC1 was observed in uncoated and coated samples (data not shown).

## 4. Discussion

Japanese plum is considered to be a climacteric fruit and rapid physiological and biochemical changes occur during its postharvest life leading to the alteration of physico-chemical traits and losses in functional compounds [30]. Furthermore, redox unbalance due to an increase of reactive oxygen species (ROS) causes oxidative stress responsible for the qualitative decay during storage [14,15].

Nowadays postharvest management aims to extend the fruits’ shelf life applying green and eco-friendly strategies along the supply chain. Chitosan-based coating represents a low-cost, eco-friendly method to control the fruit quality of whole and fresh-cut fruit along the postharvest value chain [15]. Chitosan coating smooths the fruits’ esocarp surface and coats the stomata, as evidenced by the scanning electron micrograph [16]. This physical barrier on the fruit surface controls the respiration and transpiration rates and water loss, modifying several physiological processes inside the fruit [14]. Chitosan-based coating slows down the increase of weight loss during storage, maintaining the plum fruits’ freshness, as reported in other studies on this fruit crop [16,17]. TSS, TA, firmness and fruit color represents the physico-chemical traits related to harvest time and ripening level [31]. The application of chitosan significantly decreases the ripening indices (TSS/TA) during CS and SL. In chitosan-coated plums it has been observed that the lower loss of firmness compared to uncoated ones could be due to the lower CO_2_ production, that causes an inhibition of cell wall-degrading enzymes’ activity, such as polygalacturonase and pectin methyl esterase responsible for the softening process and as suggested by Manganaris et al. [30]. Peel color changed during storage conditions but chitosan coating delayed these colorimetric changes, probably due to a delay in the rate of ripening leading to the inhibition of anthocyanin synthesis, as reported in other studies [14,16,17].

Bioactive compounds’ content, as well as polyphenol and flavonoids, increased gradually and continuously with the increase in storage period with higher values in the chitosan coated fruit, suggesting that chitosan treatment delays fruit senescence and enhances the phytochemical content and antioxidant activity during storage [16,32]. Furthermore, chitosan coating induced the changes in O_2_ and CO_2_ transmission rate through the coating layer reducing the respiration rate of the coated fruit and preserving the ascorbic acid oxidation due to ascorbic-acid oxidase and PPO activity whose activities directly depend on O_2_ concentration [33]. Retention of ascorbic acid has been reported in CEM- and PEC-coated European plums and CMC-coated Japanese plums during storage time [10,11,34]. Similar results were obtained in this study and the lower PPO activity in coated fruit samples could also be related to the ascorbic acid preservation during storage time.

Fruit ripening is a complex, genetically programmed process where non-enzymatic and enzymatic antioxidant systems play an important role to maintain reactive oxygen species at steady-state level avoiding oxidative stress. Furthermore, the generation of ROS in response to postharvest management such as cold storage and edible coatings in several fruit has been reported [14,15,35,36].

The results of this study confirmed that chitosan coating improved the activity of enzymes involved in the antioxidant defense system such as SOD and APX. In a study of ‘Sanhuali’ Japanese plum fruit it was registered that combined ascorbic acid and chitosan coating increased the SOD, POD and CAT activity [17]. Furthermore, as reported by Singh and Singh [36], the concerted action of antioxidant enzymes can also improve the acquisition of chilling tolerance in Japanese plums. High APX activity in chitosan-coated fruit could be due to the higher availability of ascorbic acid content as suggested in other fruit crops [18,32,37].

Browning is related to fruit senescence and it is due to the oxidation of phenolic substrates mediated by PPO or to the GPX activity that catalyzes single-electron oxidation of several antioxidant compounds in the presence of hydrogen peroxide [38]. Loss of membrane integrity due to disintegration and decompartmentalization occurs during the ripening progress [15].

Chitosan treatment reduced the PPO activity due to a low O_2_ availability in the plum fruit. Chitosan-coated fruit showed significantly lower GPX activity, probably as a result of delayed cell membrane damage occurring in response to low temperatures and chitosan treatment.

Postharvest oxidative stress causes the loss of membrane integrity, and MDA content and EL values are two key indicators to evaluate membrane injury that results from the lipid peroxidation process during storage [39]. The maintenance of membrane integrity in chitosan-coated fruit was confirmed by a lower MDA content and EL value. A reduction of lipid peroxidation was confirmed by low LOX activity. Chitosan coating delays ROS production preserving cell membrane structure and compartmentalization in several fruit crops [15].

The reduction in skin and flesh color changes are due to the preservation of membrane integrity and separation of PPO and GPX enzymes from their phenolic substrates. Similarly inhibited GPX and PPO activities have been observed in response to alternative technologies to chitosan coating employed on different fruit to improve their postharvest life [28,38,40,41].

## 5. Conclusions

Japanese plums due to their perishable nature have a limited shelf life. Edible coatings represent an effective postharvest technique to prolong shelf life of several fruit crops. In this study, chitosan coating formed a barrier on fruit surface, which delayed qualitative decay improving the physico-chemical traits and bioactive compounds content during cold storage and shelf life in Japanese plum fruit. Furthermore, it improves bioactive compounds content with antioxidant properties and enzymatic antioxidant defense system, and consequently it controls lipid peroxidation responsible for the loss of membrane integrity in the fruit tissues and enzymatic browning.

Chitosan-based coating is an effective preservative tool to maintain the quality, acceptability and nutritive value and thus exhibiting a huge potential for extending the postharvest life of Japanese plums during cold storage. Future research should be focused on the industrial scale application of the natural coatings to satisfy consumer demands and to reduce food losses through the supply chain.

## Figures and Tables

**Table 1:** Colorimetric parameters (L*: lightness; C: Chroma; H: Hue angle), total soluble solids (TSS: °Brix) and titratable acidity (TA; g malic acid per L juice) at harvest (0) and after 7, 14, 21, 28 and 35 days of cold storage followed by 3 days of shelf life for chitosan-coated (Chitosan) and uncoated fruit (Control).

Treatment	Time (Days)	L*	C	H	TSS	TA
	Cold Storage (CS)					
Control	0	27.27 ± 0.60 hA	9.10 ± 0.52 fB	22.75 ± 0.66 hB	12.70 ± 0.20 aA	7.20 ± 0.36 gB
	7	25.43 ± 0.55 fB	7.78 ± 0.51 eA	21.20 ± 0.55 fgAB	13.20 ± 0.21 abAB	6.80 ± 0.25 fB
	14	23.33 ± 0.51 dA	6.67 ± 0.60 dA	20.31 ± 0.47 eB	13.57 ± 0.49 bcB	4.74 ± 0.10 dB
	21	21.25 ± 0.72 cA	5.90 ± 0.40 cA	18.71 ± 0.51 cdB	14.53 ± 0.47 efB	3.52 ± 0.33 bB
	28	19.33 ± 0.50 bA	4.81 ± 0.53 bA	17.56 ± 0.29 bB	15.37 ± 0.38 gB	3.14 ± 0.13 aB
	35	18.20 ± 0.50 aA	3.73 ± 0.61 aA	16.06 ± 0.35 aB	16.47 ± 0.40 hB	3.09 ± 0.16 aB
Chitosan	0	27.29 ± 0.56 hA	9.11 ± 0.50 fB	22.79 ± 0.79 hB	12.77 ± 0.12 aA	7.16 ± 0.19 gB
	7	26.45 ± 0.57 gA	8.60 ± 0.53 fB	21.91 ± 0.58 gA	12.73 ± 0.36 aA	7.10 ± 0.06 fgAB
	14	24.49 ± 0.45 eB	7.83 ± 0.45 eB	21.12 ± 0.65 fA	13.10 ± 0.36 abAB	5.64 ± 0.14 eA
	21	22.69 ± 0.88 dB	6.78 ± 0.56 dB	19.96 ± 1.01 eA	13.73 ± 0.21 cdA	4.32 ± 0.20 cA
	28	20.64 ± 0.66 cB	5.55 ± 0.42 cB	18.82 ± 0.29 dA	14.27 ± 0.30 deA	4.02 ± 0.30 cA
	35	19.53 ± 0.49 bB	4.74 ± 0.74 bB	18.04 ± 0.31 bcA	15.03 ± 0.25 fgA	4.01 ± 0.19 cA
	**Shelf life (SL)**					
Control	0	27.27 ± 0.60 hA	9.10 ± 0.52 fA	22.75 ± 0.66 hA	12.70 ± 0.20 aA	7.20 ± 0.36 gA
	7	25.43 ± 0.55 fA	7.78 ± 0.51 eA	21.20 ± 0.55 fgAB	13.20 ± 0.21 abAB	6.80 ± 0.25 fAA
	14	23.33 ± 0.51 dA	6.67 ± 0.60 dA	20.31 ± 0.47 eA	13.57 ± 0.49 bcB	4.74 ± 0.10 dA
	21	21.25 ± 0.72 cA	5.90 ± 0.40 cA	18.71 ± 0.51 cdA	14.53 ± 0.47 efB	3.52 ± 0.33 bA
	28	19.33 ± 0.50 bA	4.81 ± 0.53 bA	17.56 ± 0.29 bA	15.37 ± 0.38 gB	3.14 ± 0.13 aA
	35	18.20 ± 0.50 aA	3.73 ± 0.61 aA	16.06 ± 0.35 aA	16.47 ± 0.40 hB	3.09 ± 0.16 aA
Chitosan	0	27.29 ± 0.56 hA	9.11 ± 0.50 fA	22.79 ± 0.79 hA	12.77 ± 0.12 aA	7.16 ± 0.19 gA
	7	26.45 ± 0.57 gB	8.60 ± 0.53 fB	21.91 ± 0.58 gB	12.73 ± 0.36 aA	7.10 ± 0.06 fgAB
	14	24.49 ± 0.45 eB	7.83 ± 0.45 eB	21.12 ± 0.65 fB	13.10 ± 0.36 abB	5.64 ± 0.14 eB
	21	22.69 ± 0.88 dB	6.78 ± 0.56 dB	19.96 ± 1.01 eB	13.73 ± 0.21 cdA	4.32 ± 0.20 cB
	28	20.64 ± 0.66 cB	5.55 ± 0.42 cB	18.82 ± 0.29 dB	14.27 ± 0.30 deA	4.02 ± 0.30 cB
	35	19.53 ± 0.49 bB	4.74 ± 0.74 bB	18.04 ± 0.31 bcB	15.03 ± 0.25 fgA	4.01 ± 0.19 cB

Means followed by the same uppercase letters depict differences between treatments, whereas lowercase letters depict differences between storage times rage/shelf life *p* = 0.05 (Duncan Test).

**Table 2:** Bioactive compounds content (TP: total phenols-mg GAE per 100 g FW; TF: total flavonoids-mg CE per 100 g FW; TAC: total anthocyanin-mg C3g per 100 g FW; AA: Ascorbic acid-mg AA per g FW) and antioxidant activity (DPPH-µmol TE per g FW) at harvest (0) and after 7, 14, 21, 28 and 35 days of cold storage followed by 3 days of shelf life for chitosan-coated (Chitosan) and uncoated fruit (Control).

Treatment	Time (Days)	TP	TF	TAC	AA	TAA
		Cold Storage				
Control	0	161.73 ± 4.88 aA	48.71 ± 1.88 aA	41.29 ± 2.49 aA	23.51 ± 0.17 gA	3.64 ± 0.35 aA
	7	172.29 ± 4.23 abA	62.39 ± 1.91 bB	48.42 ± 2.15 bA	20.59 ± 0.46 eA	4.02 ± 0.49 abAB
	14	188.92 ± 7.19 bcA	76.57 ± 9.12 cA	54.97 ± 1.05 cB	18.52 ± 0.30 dA	4.56 ± 0.60 bcAB
	21	192.05 ± 9.82 bcA	82.10 ± 10.81 cAd	68.48 ± 0.58 eB	16.90 ± 0.63 cA	5.61 ± 0.35 deAB
	28	202.55 ± 11.96 cdA	98.52 ± 7.07 eA	77.22 ± 0.19 fB	15.81 ± 0.25 bA	6.07 ± 0.46 efA
	35	213.64 ± 5.39 dA	105.06 ± 2.27 eA	84.80 ± 2.80 gB	14.36 ± 0.37 aA	6.69 ± 0.17 fgA
Chitosan	0	162.74 ± 5.43 aA	48.80 ± 1.25 aA	41.62 ± 1.93 aA	23.48 ± 0.20 gA	3.51 ± 0.18 aA
	7	187.40 ± 5.93 bcB	76.48 ± 2.96 cB	45.88 ± 1.98 bA	22.04 ± 0.76 fB	5.17 ± 0.34 cdB
	14	201.83 ± 14.41 cdB	85.65 ± 6.10 cdB	47.99 ± 1.28 bA	20.56 ± 0.17 eB	6.03 ± 0.29 efB
	21	234.63 ± 20.54 eB	93.60 ± 10.20 deB	57.30 ± 0.42 cA	18.87 ± 0.60 dC	6.57 ± 0.40 fgB
	28	268.74 ± 19.87 fB	105.47 ± 5.53 eA	63.12 ± 4.27 dA	17.04 ± 0.14 cB	7.11 ± 0.20 ghB
	35	295.32 ± 12.63 gB	126.44 ± 9.59 fA	76.95 ± 2.85 f	16.60 ± 0.56 cB	7.68 ± 0.58 h
**Shelf life**						
Control	0	159.49 ± 2.17 aA	46.83 ± 5.91 aA	39.80 ± 0.55 aA	22.20 ± 0.22 efAB	3.41 ± 0.15 aA
	7	162.64 ± 6.18 aA	58.19 ± 8.03 bcAB	47.71 ± 5.44 bcAB	18.87 ± 0.59 dA	3.88 ± 0.04 bA
	14	178.15 ± 10.41 abA	62.74 ± 6.24 cA	52.20 ± 0.68 cdAB	16.31 ± 0.26 cB	4.09 ± 0.13 bcA
	21	187.20 ± 5.07 bA	75.54 ± 8.30 dA	65.96 ± 1.76 eB	14.40 ± 0.41 bB	4.90 ± 0.01 dA
	28	194.31 ± 16.04 bA	82.77 ± 7.99 dA	75.72 ± 5.37 fB	13.49 ± 0.21 bB	5.77 ± 0.11 eA
	35	196.51 ± 11.56 bA	97.77 ± 3.65 eA	82.72 ± 1.64 gB	12.12 ± 0.88 a	6.03 ± 0.16 efA
Chitosan	0	176.88 ± 10.09 abAB	47.82 ± 1.77 abA	40.67 ± 1.58 aA	22.64 ± 0.65 fB	3.56 ± 0.16 aA
	7	179.89 ± 7.84 abAB	73.39 ± 2.27 dB	44.37 ± 2.71 abA	21.74 ± 1.53 eB	4.27 ± 0.04 cB
	14	186.84 ± 11.03 bA	78.17 ± 5.20 dA	46.92 ± 3.37 bcA	18.83 ± 0.09 dA	5.78 ± 0.04 eB
	21	200.95 ± 25.82 bA	82.12 ± 7.51 dA	55.18 ± 2.39 dA	16.37 ± 0.18 cA	6.15 ± 0.19 fB
	28	230.87 ± 6.46 cB	98.31 ± 6.25 eB	61.92 ± 4.00 eA	15.60 ± 0.36 cA	6.73 ± 0.18 gB
	35	269.74 ± 14.02 dB	115.72 ± 7.56 fB	74.23 ± 2.27 fA	14.30 ± 0.92 bA	7.13 ± 0.40 hB

Means followed by the same uppercase letters depict differences between treatments, whereas lowercase letters depict differences between storage times rage/shelf life *p* = 0.05 (Duncan Test).

**Table 3:** Activity of guaiacol peroxidase (GPX; µmol guaiacol per mg protein), polyphenol oxidase (PPO; mmol per mg protein) and lipoxygenase (LOX; mmol per mg protein), malondialdehyde content (MDA; nmol per g FW) and electrolyte leakage (EL;%) at harvest (0) and after 7, 14, 21, 28 and 35 days of cold storage followed by 3 days of shelf life for chitosan-coated (Chitosan) and uncoated fruit (Control).

Treatment	Time (Days)	GPX	PPO	LOX	MDA	EL
		Cold Storage				
Control	0	0.48 ± 0.04 deA	3.66 ± 0.20 aA	127 + 54 ± 15.62 aA	8.85 0.30 aA	35.90 ± 0.94 aA
	7	0.84 ± 0.03 gB	4.40 ± 0.47 abcB	146.25 ± 20.40 abB	11.01 ± 1.45 cB	45.71 ± 1.33 cB
	14	1.70 ± 0.10 iB	5.18 ± 0.59 cdeB	150.20 ± 10.02 abB	14.24 ± 0.76 eB	56.37 ± 1.48 eB
	21	0.63 ± 0.01 fB	5.50 ± 0.43 efB	155.84 ± 16.49 bB	14.84 ± 0.19 eB	65.92 ± 0.65 fB
	28	0.44 ± 0.02 dB	6.00 ± 0.19 fgB	160.56 ± 10.86 bB	16.91 ± 0.30 fB	76.75 ± 1.0 hB
	35	0.24 ± 0.01 bB	6.36 ± 0.21 gB	225.38 ± 18.31 dB	18.97 ± 0.65 gB	83.59 ± 3.09 iB
Chitosan	0	0.48 ± 0.04 deA	3.70 ± 0.10 aA	128.22 ± 15.62 aA	8.79 ± 0.38 aA	35.71 ± 1.31 aA
	7	0.65 ± 0.07 fA	3.82 ± 0.14 aA	128.76 ± 12.80 aA	9.43 ± 0.37 abA	37.19 ± 1.66 aA
	14	1.14 ± 0.08 hA	4.03 ± 0.33 abA	137.16 ± 13.37 abA	9.87 ± 0.06 abA	40.97 ± 2.17 bA
	21	0.54 ± 0.01 eA	4.34 ± 0.16 abcA	139.57 ± 15.73 abA	10.22 ± 0.29 bcA	51.49 ± 1.55 dA
	28	0.33 ± 0.03 cA	4.54 ± 0.49 abcA	149.02 ± 2.84 abA	12.48 ± 0.73 dA	65.75 ± 2.11 fA
	35	0.17 ± 0.03 aA	4.86 ± 0.59 bcdA	189.41 ± 2.12 cA	13.78 ± 0.42 eA	70.61 ± 2.56 gA
		**Shelf life**				
Control	0 + 3	0.74 ± 0.03 efB	4.55 ± 0.03 abB	233.49 ± 19.04 dB	9.89 ± 0.77 abB	36.84 ± 0.34 aA
	7 + 3	0.94 ± 0.03 gB	5.05 ± 0.53 bcB	239.21 ± 9.79 deB	12.90 ± 0.57 cdB	47.28 ± 1.07 dB
	14 + 3	1.71 ± 0.18 iB	5.77 ± 0.49 deB	247.72 ± 22.41 defB	16.83 ± 0.36 efB	58.93 ± 1.36 fB
	21 + 3	0.80 ± 0.08 fB	6.32 ± 0.34 efB	264.13 ± 15.08 efB	17.26 ± 1.50 efB	68.36 ± 0.85 gB
	28 + 3	0.50 ± 0.07 cdB	6.56 ± 0.08 fB	271.08 ± 2.39 fB	18.99 ± 0.56 fgB	78.20 ± 1.79 iB
	35 + 3	0.74 ± 0.03 efB	6.66 ± 0.32 fB	351.06 ± 10.88 hB	20.55 ± 1.16 gB	86.36 ± 1.10 lB
Chitosan	0 + 3	0.74 ± 0.02 efA	3.91 ± 0.44 aA	134.62 ± 0.65 aA	8.31 ± 1.02 aA	36.70 ± 1.13 aA
	7 + 3	1.27 ± 0.07 hA	3.97 ± 0.19 aA	168.64 ± 13.29 bA	10.20 ± 1.34 abA	39.40 ± 0.87 bA
	14 + 3	0.66 ± 0.02 eA	4.30 ± 0.54 aA	201.74 ± 13.61 cA	11.64 ± 0.99 bcA	43.30 ± 0.85 cA
	21 + 3	0.40 ± 0.04 bcA	4.48 ± 0.22 abA	246.25 ± 10.50 defA	12.78 ± 1.75 cdA	54.59 ± 1.37 eA
	28 + 3	0.23 ± 0.02 aA	5.12 ± 0.19 bcdA	258.50 ± 1.90 defA	14.08 ± 1.46 cdA	67.40 ± 0.96 gA
	35 + 3	0.74 ± 0.02 efA	5.72 ± 0.26 cdeA	306.56 ± 14.69 gA	15.11 ± 1.44 deA	74.29 ± 0.89 hA

Means followed by the same uppercase letters depict differences between treatments, whereas lowercase letters depict differences between storage times rage/shelf life *p* = 0.05 (Duncan Test).

## Data Availability

The Authors did not report any data.
